# Improved Artificial Potential Field Algorithm Assisted by Multisource Data for AUV Path Planning

**DOI:** 10.3390/s23156680

**Published:** 2023-07-26

**Authors:** Tianyu Xing, Xiaohao Wang, Kaiyang Ding, Kai Ni, Qian Zhou

**Affiliations:** Division of Advanced Manufacturing, Shenzhen International Graduate School, Tsinghua University, Shenzhen 518055, China

**Keywords:** autonomous underwater vehicles (AUVs), deep reinforcement learning (DRL), improve artificial potential field, path planning

## Abstract

With the development of ocean exploration technology, the exploration of the ocean has become a hot research field involving the use of autonomous underwater vehicles (AUVs). In complex underwater environments, the fast, safe, and smooth arrival of target points is key for AUVs to conduct underwater exploration missions. Most path-planning algorithms combine deep reinforcement learning (DRL) and path-planning algorithms to achieve obstacle avoidance and path shortening. In this paper, we propose a method to improve the local minimum in the artificial potential field (APF) to make AUVs out of the local minimum by constructing a traction force. The improved artificial potential field (IAPF) method is combined with DRL for path planning while optimizing the reward function in the DRL algorithm and using the generated path to optimize the future path. By comparing our results with the experimental data of various algorithms, we found that the proposed method has positive effects and advantages in path planning. It is an efficient and safe path-planning method with obvious potential in underwater navigation devices.

## 1. Introduction

In recent years, thanks to the abundance of marine resources, the “blue industry” has received widespread attention and flourished. However, due to the development of technology and the unknown as well as complex environment, 95% of the ocean remains unexplored. Autonomous underwater vehicles (AUVs) have been widely used in environmental observation, resource exploration, biological surveying, auxiliary positioning, and other underwater tasks by virtue of their autonomy and maneuverability [1,2,3]. In order to meet the needs of marine development, strict requirements have been proposed for the automation of AUVs. The core technology of AUV autonomy is to complete path planning and obstacle avoidance [4], which determine the application prospects of AUVs.

Owing to the complexity and variability of the marine environment, underwater path planning is a complex issue that has received widespread attention. The current means of AUV underwater path planning mainly include the ant colony algorithm [5,6], fuzzy algorithm [7], genetic algorithm [8,9], algorithms based on neural networks [10], and algorithms based on the artificial potential field theory [11,12]. Zhang et al. [13] used a combination of the ant colony algorithm and forward sonar to accomplish two-dimensional obstacle avoidance. Dong et al. [14] implemented a path-planning method for AUVs, achieving a smoother generated path. Somaiyeh et al. [15] combined the ant colony algorithm with the energy of an AUV to complete the path-planning task with minimal energy. Chen et al. [16] proposed a method combining a neural network with path planning, and found that robots can make decisions by imitating human thinking during navigation. Based on local environmental information and obstacle features, robots can make decisions by simulating human beings, collecting all of the information necessary to build a database and training the database through supervised learning methods to generate an optimal neural network model. The experimental results show that the proposed algorithm enables robots to achieve excellent obstacle avoidance ability in the path-planning task.

The above algorithm only focuses on path planning in a 2D environment, but the practical applications are more focused on the 3D scene, so research should focus on 3D environments. Ma et al. [5] introduced an alarm pheromone into the ant colony algorithm (AP-ACO) and applied the new algorithm to the path planning of an AUV in complex underwater environments. Experimental results show that AP-ACO has the advantages of faster convergence speed and stability compared with the ordinary ACO algorithm, but the algorithm works less than ideally in unknown environments. Li Bin et al. [17] designed an improved sparrow algorithm and introduced an adaptive weighted silver balancing strategy to improve the convergence rate and search ability of the algorithm. The proposed algorithm completes the global path planning of AUVs in complex marine environments subject to many influencing factors, but the turning angle is large. Wei et al. [18] proposed a novel super-heuristic algorithm based on an evolutionary strategy; the proposed algorithm combined a metaheuristic framework with a selection function to evaluate the performance of low-level heuristic operators online. This online learning algorithm has better computational efficiency and robustness and achieves satisfactory performance in path planning in complex marine environments, although it is difficult to apply. Recently, with the rapid development of information technology, artificial intelligence has made considerable breakthroughs in technological and application fields [19]. Deep learning (DL) [20] and reinforcement learning (RL) [21] have made rapid progress with the support of artificial intelligence technology, and have shown great advantages in their respective fields. DL achieves significant advantages in perception ability due to its neural network structure, and RL helps to make optimal action decisions by maximizing the value function. Mnih, V et al. [22] proposed a deep Q-network (DQN) algorithm, which skillfully combines DL and RL together and initiates an era of deep reinforcement learning (DRL). DRL combines the advantages of DL and RL [23], and it can provide a more perfect solution in response to perception decision problems in complex systems. Silver [24] proposed a deterministic policy gradient (DPG) algorithm. Compared with the stochastic policy gradient algorithm, the performance of the proposed algorithm in high-latitude action space has significant advantages. Lillicrap, T. Page et al. [25] proposed a deep deterministic policy gradient (DDPG) algorithm. The DDPG can easily deal with complex problems and large network structures because of its powerful functions, and the algorithm is simple and convergent. Wei et al. [26] successfully combined the DDPG algorithm with an underwater glider to realize a two-dimensional obstacle avoidance function in the underwater glider under the control of DRL in the face of time-varying ocean currents. Chu et al. [27] inserted a dynamic reward function into the DDQN algorithm, enabling AUVs to simultaneously complete the tasks of path planning and obstacle avoidance in complex underwater environments. Fang et al. [28] controlled the attitude of an AUV during navigation using the DDPG algorithm and solved the control fault problem at the critical value of the yaw angle. Yang et al. [29] proposed a path-planning algorithm based on near-end strategy optimization; the proposed algorithm combines a deep reinforcement learning network with the features of local obstacles and selects the optimal strategy according to the environmental information. The results show that the paths generated by this algorithm are time-saving and collision-free in complex underwater environments. Wang et al. [30] proposed an algorithm based on a simplified deep deterministic policy gradient in order to optimize the complex nonlinear problem of AUVs during navigation. The algorithm simplifies the training process of the neural network and optimizes the path of an AUV. The abovementioned method is designed for the ideal operating environment of the AUVs and may not achieve satisfactory results in actual underwater operation experiments, so attention should be paid to the sensing equipment carried by an AUV itself. Bu et al. [31] used sensing devices for communication and combined the DRL algorithm with the energy of an AUV to generate an optimal operating path; however, the obstacle avoidance ability was not ideal. Cao et al. [4] acquired image information through the forward sonar of an AUV and combined the graphic information with the DRL algorithm to effectively avoid obstacles in the face of complex underwater environments, planning a reasonable forward path, although path optimization is not implemented. The possibility of multiple-sensor devices jointly assisting AUVs in path planning has attracted increasing attention.

This paper presents an improved artificial potential field method with overcoming local minima. When an AUV is trapped in the local optimal trap, all the distance data at this time are introduced into the artificial potential field, such that the AUV trapped in the local optimal trap escapes from the trap. In path planning, the improved artificial potential field method is combined with DRL, and the distance data are used to optimize the reward function to generate a safe path. For reducing the extreme turn events of an AUV at run time, the generated path is used to optimize the future path.

In this paper, Section 2 presents the motion model. Section 3 introduces the methods used, including the improvement of an artificial potential field, the utilization of gyroscope data, and the use of DRL. Section 4 shows the experimental results, which intuitively show the advantages of the method proposed in this paper through data analysis and comparison. Finally, the conclusions and future work are given in Section 5.

## 2. Problem Formulation

**Figure 1:**
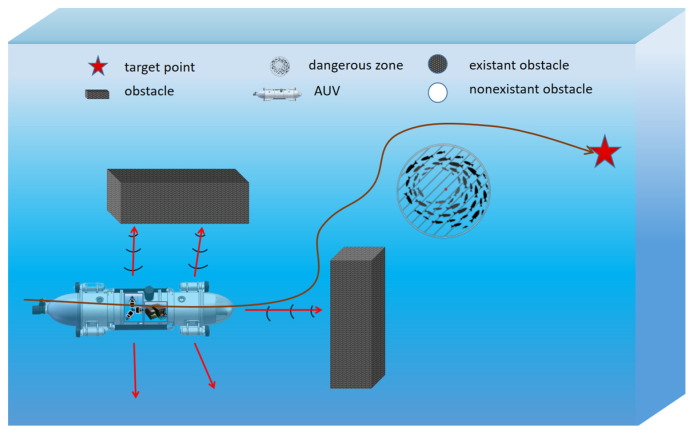
Schematic diagram of the underwater operation of an AUV with multiple sensors.

Figure 1 shows a schematic of an AUV with multiple sensors during underwater path planning. In the case of simulated deep AUV operation, the AUV can successfully avoid obstacles to reduce the occurrence of peak steering, and the planned path is smooth.

**Figure 2:**
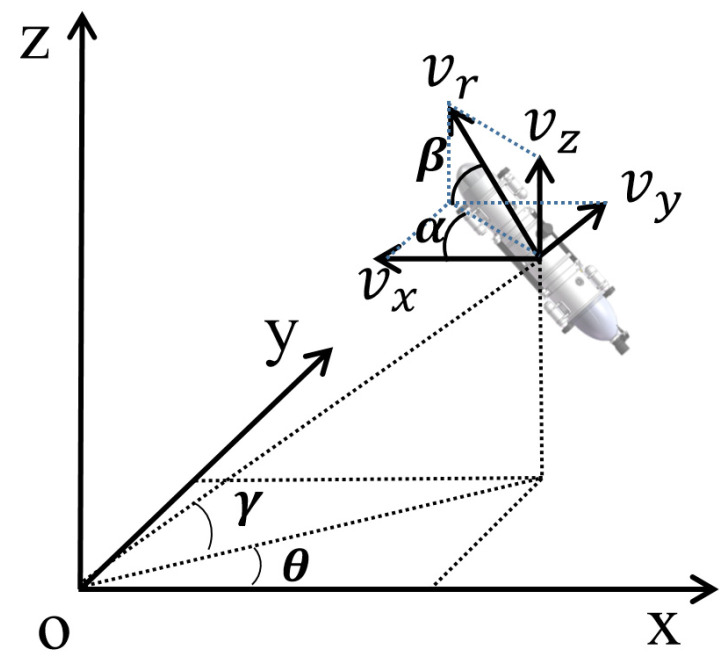
Kinematic and dynamic models of the AUV.

In the process of AUVs performing tasks, most of them take place in underwater scenes. In a static environment and without the loss of generality, only the path-planning problem for the main 3-DOF underactuated AUV is considered. Figure 2 shows the kinematic and dynamic model of an AUV. According to the method proposed by T. I. Fossen et al. [32], the motion of AUV swaying, pitch, and roll is ignored. Therefore, the AUV kinematics and dynamics are expressed as follows:

(1) $$\dot{\eta }=R_{y}(\alpha )*R_{z}(\beta )*v_{r,}$$

(2) $$M{\dot{v}}_{r}+C(v_{r})v_{r}+D(v_{r})v_{r}=G(v_{r})*\tau ,$$

(3) $$\mathrm{where}\;R_{y}(\alpha )=\left(\begin{array}{ccc} \cos\alpha & 0 & -\sin\alpha \\ 0 & 1 & 0\\ \sin\alpha & 0 & \cos\alpha \end{array}\right),\;R_{z}(\beta )=\left(\begin{array}{ccc} \cos\beta & \sin\beta & 0\\ -\sin\beta & \cos\beta & 0\\ 0 & 0 & 1 \end{array}\right).$$

To explain the above formula clearly and concisely, two reference frames need to be assumed: The fixed-Earth inertial coordinate system {i} and the fixed-body inertial coordinate system {b}. $\eta ={\left[x,\;y,\;z\;\right]}^{T}$ depicts the positional status of the AUV in coordinate system {i}. $v_{r}={\left[v_{r},\;\theta ,\;\gamma \;\right]}^{T}$ represents the velocity matrix of the AUV, where $v_{r}$ represents the forward velocity of the AUV. θ and γ represent the yaw and pitch in coordinate system {i}, respectively. Matrices $R_{y}(\alpha )$ and $R_{z}(\beta )$ convert the vectors in coordinate system {b} into the vectors in coordinate system {i}. The $M\in R^{3*3}$ matrix is the mass matrix, and the $C(v_{r})\in R^{3*3}$ matrix represents the Coriolis centripetal matrix. The $D(v_{r})\in R^{3*3}$ matrix represents the damping matrix, and $G(v_{r})\in R^{3*3}$ is the input configuration matrix. $\tau ={\left[\tau_{v},\;\tau_{\alpha },\tau_{\beta }\;\right]}^{T}$ represents three independent input control signals, where $\tau_{v}$ represents the thrust of the propeller, $\tau_{\alpha }$ represents the real-time steering angle, and $\tau_{\beta }$ represents the real-time attack angle. Usually, the propeller thrust is constant, and the steering and attack angles are limited; thus, $|\tau_{\alpha }|\leq \tilde{\tau_{\alpha }}$ and $|\tau_{\beta }|\leq \tilde{\tau_{\beta }}$, where $\tilde{\tau_{\alpha }}$ and $\tilde{\tau_{\beta }}$ represent the saturated boundaries of $\tau_{\alpha }$ and $\tau_{\beta }$, respectively.

## 3. Method

### 3.1. Utilization of Multisource Data

There are many sensors in an AUV, and their data are helpful for AUVs in path planning. In this paper, multisource data used are derived from a gyroscope and rangefinder. Gyroscopes are widely used in AUVs, which can assist AUVs in navigation and positioning [33]. Based on the structure and kinematic constraints of an AUV, the gyroscope can reflect the motion information of an AUV in real time [34]. The gyroscope data are processed by Kalman filtering, which can make the motion path smoother and protect the mechanical structure of an AUV [35].

When an AUV is moving underwater, only the attack angle and steering angle are usually considered. Considering its mechanical properties and actual operation state, it is necessary to restrict the attack angle, steering angle, and pitch angle. The steering angle value size cannot exceed the saturated boundary. Similarly, the attack angle and pitch angle must not exceed the corresponding saturated boundaries.

Since an AUV’s navigation angle (steering angle and attack angle) is determined by the force at its current position, it is highly possible that AUVs will dramatically change their direction. When this case happens, AUVs may not be able to complete the angle conversion. Therefore, in the simulation process of path planning, the mechanical performance factor of an AUV must be considered. The gyroscope can measure the size of the steering angle and attack angle in real time, and perform Kalman filtering on the AUV’s steering angle and attack angle to achieve the purpose of optimizing the navigation angle and safe navigation.

A Kalman filter is an optimal estimator. It uses a recursive method to update the state output of the model by inputting the recent measurements into a linear model. In a Kalman filter, the state transition equation and the target observation equation are as follows:

(4) $$x_{k+1}=A_{k}x_{k}+B_{k}u_{k}+w_{k,}$$

(5) $$y_{k}=H_{k}x_{k}+v_{k.}$$

Equation (4) is the model function that transfers state x from step k to step k+1, where $x_{k}\in R^{n}$ is the state of the system, $x_{k}$ is the actual state vector after measurement, and $x_{k+1}$ is the next state vector estimated by the equation. $A_{k}\in R^{n*n}$ is the transition matrix, while $B_{k}$ is a linear matrix that transforms the dimension of the residual vector, $u_{k}$, into the state vector, $x_{k}$. $u_{k}$ is the “forcing” term in the model in the form of a linear residual. Let $w_{k}$ be the model estimation error. Equation (5) is the observation function that relates the state vector, $x_{k}$, to the observation vector, $y_{k}$, where $H_{k}$ is the linear observation operator (ensuring the linearity of the system) and $v_{k}$ is the observation error.

(6) $$E[v_{i}v_{j}^{T}]=r\;\forall \;i=j\in N,$$

(7) $$E[w_{i}w_{j}^{T}]=Q\;\forall \;i=j\in N.$$

In Equation (6), r is the covariance matrix of the observation error, $v_{k}$, while Q is the covariance matrix of $w_{k}$ in Equation (7).

**Figure 3:**
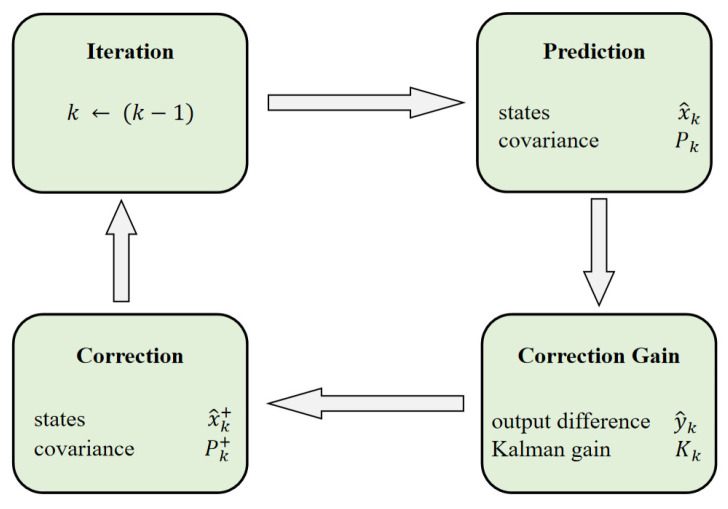
The principle of a Kalman filter.

In Figure 3, the principle of Kalman filtering is shown [36,37]. Kalman filtering is a predictor–corrector method. Firstly, a state, ${\hat{x}}_{k}$, and its corresponding covariance matrix, $P_{k}$, are predicted. Secondly, the Kalman gain, $K_{k}$, for this stage is calculated. Then, the difference between the real measurement result, $u_{k}$, and the predicted measurement result, $y_{k}$, is weighted by $K_{k}$ to correct the prediction, ${\hat{x}}_{k}^{+}$ and $P_{k}^{+}$. Finally, ${\hat{x}}_{k}$ is updated to complete the initialization.

### 3.2. Improved Artificial Potential Field (IAPF) Algorithm

In 1986, Khatib [38] innovatively proposed a new algorithm of virtual force, the artificial potential field (APF) method. This method manually simulates an electric potential field similar to the form of an electromagnetic field in the working scene. The robot is subjected to both gravitational and repulsive forces in the scene and plans its path according to the characteristics of the potential field.

The artificial potential field method has some outstanding advantages, such as a small amount of calculation, easy construction of the model, and remarkable effect of dynamic obstacle avoidance; however, it still has shortcomings. For example, it is easy for the robot to fall into the local optimum and sometimes cannot reach the target point. When the integrated potential field of the target point is not the minimum value in the whole integrated potential field, the case of an unreachable target point will be generated [39]. The motion of the robot in the scene is affected by the resultant force formed by the superposition of attractive forces and repulsive forces, and the robot determines the direction and magnitude of the next movement under the action of the resultant force. If the resultant force is zero, the robot will stop moving. The more obstacles there are that repel the robot at the same time, the higher the probability of the resultant force being zero. This condition is defined as the “potential field trap” in this paper.

#### 3.2.1. Improved Method for Unreachable Target Point Problem

The essence of the unreachable target point problem is that the integrated potential field of the target point is not the minimum value in the whole integrated potential field. In order to overcome the unreachable problem of the target point in the APF, a distance correction function is added to the repulsive potential field, while the attractive potential field remains unchanged. The distance correction function can balance the changes in repulsive and attractive forces, especially in the case of a rapidly increasing repulsive potential field. Thus, when a robot approaches the target point, the correction function can be added to ensure that the target point is at the minimum value in the integrated potential field. The new repulsive potential field function is as follows:

(8) $$U_{rep}=\left\{\begin{array}{cc} \frac{1}{2}k_{rep}{\left(\frac{1}{\left(X_{n}-X_{0}\right)}-\frac{1}{r_{0}}\right)}^{2}d^{n}(X_{n},X_{g})\;, & (X_{n}-X_{0})\leq r_{0}\\ 0, & (X_{n}-X_{0})>r_{0}. \end{array}\right.$$

$d^{n}(X_{n},X_{g})$ is the Euclidean distance from the current position to the target point raised to the power of *n*, and *n* is any real number greater than zero. According to Equation (8), when the robot approaches the target point, the repulsive potential field will tend to zero, so that the integrated potential field at the target point is still the minimum.

The repulsive force is the negative gradient force of the new repulsive potential field, which is mathematically expressed as follows:

(9) $$F_{rep}=-\nabla U_{rep}=\left\{\begin{array}{cc} F_{rep1}+F_{rep2}\;, & (X_{n}-X_{0})\leq r_{0}\\ 0 & (X_{n}-X_{0})>r_{0}, \end{array}\right.$$

where *F_rep_*_1_ and *F_rep_*_2_ are as follows:

(10) $$F_{rep1}=k_{rep}(\frac{1}{\left(X_{n}-X_{0}\right)}-\frac{1}{r_{0}})\frac{d^{n}(X_{n}-X_{g})}{d^{2}(X_{n}-X_{0})},$$

(11) $$F_{rep2}=\frac{n}{2}k_{rep}{\left(\frac{1}{\left(X_{n}-X_{0}\right)}-\frac{1}{r_{0}}\right)}^{2}d^{n-1}(X_{n},X_{g}).$$

#### 3.2.2. Improved Method for Potential Field Trap Problem

In the optimal scenario, an AUV would come to a stop upon reaching its intended destination; however, when an AUV navigates through an integrated potential field comprising numerous obstacles, it may encounter situations where the resultant force acting on it becomes zero, or the integrated potential field at its current position reaches a minimum value. In such instances, the AUV becomes motionless and is unable to progress towards the goal point, ultimately impeding its ability to reach the intended destination.

When an AUV is attempting to escape a “potential field trap,” it is crucial to prioritize both obstacle avoidance and efficient path planning. In a study by Zhou et al. [40], they propose an improved method that primarily focuses on accomplishing the path-planning task while neglecting obstacle collision avoidance. To effectively address the issue of potential field traps, it is essential to leverage all of the distance data provided by an AUV’s rangefinders. Rangefinders, such as sonar and photoacoustic rangefinders [41], are employed to continuously monitor the closest distance between an AUV and surrounding obstacles in real time, ensuring the safe navigation of an AUV. The accuracy of the ranging measurements directly impacts the safety of AUV navigation and facilitates smoother navigation, enabling AUVs to escape potential field traps more efficiently.

**Figure 4:**
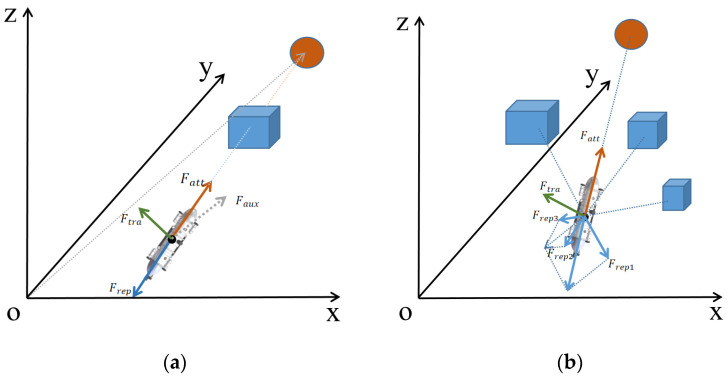
The improvement of the AUV moving strategy in the case of potential field traps. The circle is the target and the square is the obstacle. (**a**) The improvement of the AUV moving strategy when there is a single obstacle. (**b**) The improvement of the AUV moving strategy when there are multiple obstacles.

In an underwater environment, when an AUV becomes trapped in a potential field, it can generally be categorized into two cases: a single obstacle or multiple obstacles. The strategy for improving the method in the case of a single obstacle is depicted in Figure 4a, while the strategy for the case of multiple obstacles is illustrated in Figure 4b.

In a scenario involving a single obstacle, an AUV can be ensnared in a potential field trap only when the AUV, obstacle, and target point align perfectly on a straight line. In this particular position, the combined effect of the repulsive force and attractive force becomes zero, leading to the entrapment of the AUV within the potential field. To overcome this situation, a traction force is employed to assist the AUV in escaping the potential field trap. Initially, an auxiliary force is established within the Cartesian coordinate system, directed from the origin to the target point, and its magnitude is set to match the attraction force between the AUV and the target point. The traction force required to free the AUV from the potential field trap is obtained through the vector product of the auxiliary force and the attractive force exerted by the target point. By employing this traction force, the AUV successfully escapes the potential field trap and reaches its intended target point.

In a scenario involving multiple obstacles, when an AUV becomes trapped in a potential field, it is crucial to select an appropriate auxiliary vector to facilitate its escape. To achieve this, the nearest obstacle to the AUV is identified using a photoacoustic rangefinder, and the repulsive force generated by this obstacle is employed as the auxiliary force. By chance, if this repulsive force aligns perfectly in magnitude and is in the opposite direction to the attractive force, the search continues for the second closest obstacle to the AUV using the photoacoustic rangefinder. The repulsive force exerted by the second closest obstacle is then utilized as the new auxiliary force. This process is repeated until a suitable auxiliary force is found. By employing this method to identify the auxiliary force, the AUV ensures safe navigation while effectively escaping the potential field trap and reaching the target point as expeditiously as possible. The traction force required for the AUV to escape the potential field trap and reach the target point smoothly is determined through the vector product of the auxiliary force and the attraction force exerted by the target point. By integrating this approach, not only can the AUV navigate safely, but it can also overcome potential field traps and swiftly reach the target point.

### 3.3. Multisource-Data-Assisted AUV Path-Planning Method Based on the DDPG Algorithm

**Figure 5:**
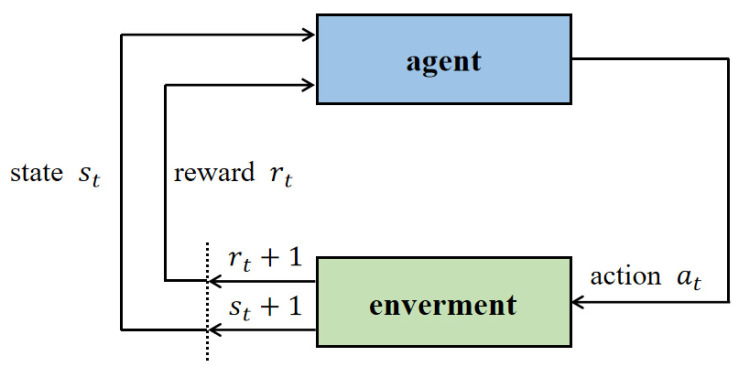
The agent interacts with the environment during the decision-making process of reinforcement learning.

Reinforcement learning, which falls under the domain of machine learning, serves as the foundation for deep reinforcement learning [42]. In this framework, an agent is trained through a reward-based system, aiming to maximize the cumulative reward while learning an optimal strategy to achieve its objectives. The agent directly interacts with the environment and learns by evaluating the resulting action values, as depicted in Figure 5. During agent–environment interaction, the agent selects the subsequent action based on the current state and the environmental reward. This action $a_{t}$ leads to a transition from state $s_{t}$ to new state $s_{t+1}$, and the agent receives a corresponding environmental reward $r_{t}$. Leveraging the current state, the agent strives to maximize the expected reward value and continually learns in addition to enhancing its action strategy throughout the interaction with the real-world environment [43].

Deep learning, as a vital component of machine learning, utilizes deep neural networks to autonomously learn from raw data and extract highly accurate features. Reinforcement learning, on the other hand, plays a significant role in evaluating actions taken by an agent during its interaction with the environment, allowing the agent to maximize rewards and acquire optimal strategies. By combining the strengths of deep learning (DL) and reinforcement learning (RL), deep reinforcement learning (DRL) has achieved remarkable advancements in continuous motion control and has been proven to be highly effective in autonomous driving systems. Within DRL, the deep deterministic policy gradient (DDPG) algorithm has emerged as a valuable solution for addressing problems within continuous motion spaces. It has gained traction and is being increasingly employed in the field of autonomous transportation, showcasing its potential and applicability in this domain.

#### 3.3.1. Deep Deterministic Policy Gradient (DDPG)

**Figure 6:**
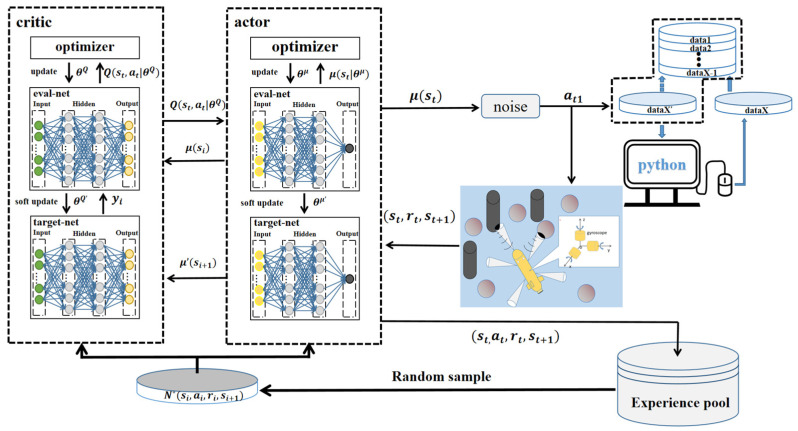
Multisource-data-assisted AUV path-planning method based on the DDPG algorithm.

Figure 6 is a multiple-sensor-assisted AUV path-planning method based on the DDPG algorithm. The DDPG algorithm is formed by the architecture of Actor–Critic and combined with the algorithm of a DQN. It both solves the continuous action problem and enhances the stability as well as effectiveness of the network training, in addition to overcoming the non-convergence problem when using the neural network to approximate the function value [44]. Its network structure includes an actor network and evaluator network, both of which have their own online network and target network. Both online networks output and evaluate actions in real time, as well as training and updating network parameters online. Its network structure includes an actor network and evaluator network, which each have their own online network and target network. The two target networks will update the value network system and the actor network system, but the network parameters in the system are not trained and updated in real time [45]. As can be seen in Figure 6, the agent outputs an action based on the current state, $s_{t}$, generated by the environment information and its own data. When the action is an effective action, it will receive a feedback reward, rt, from the environment. The data containing the current state information, action, reward, and next action information will then be stored in the experience pool. At the same time, the neural network will extract sample data from the experience pool for training and realize the accuracy as well as stability of the algorithm by adjusting the action strategy.

The critical network updates the network parameters by minimizing the loss function, $L(\theta^{Q})$:

(12) $$\left\{\begin{array}{c} L(\theta^{Q})=\frac{1}{N}\sum_{i}{\left(y_{i}-Q(s_{i},a_{i}|\theta^{Q})\right)}^{2}\\ y_{i}=r(s_{i},a_{i})+\gamma Q^{\prime }(s_{i+1},\mu^{\prime }(s_{i+1}|\theta^{\mu^{\prime }})|\theta^{Q^{\prime }}), \end{array}\right.$$

where $\theta^{Q}$ and $\theta^{Q^{\prime }}$, respectively, represent the parameters of the evaluation network and the target network. $\theta^{\mu^{\prime }}$ represents the parameters of the target network in the actor network and N is the number of experiences learned.

Updates the current network parameters for the actor:

(13) $$\nabla_{\theta_{\mu }}J=\frac{1}{N}\sum_{i}^{N}\nabla_{a_{i}}Q(S_{i},a_{i}|\theta^{Q})\nabla_{\theta^{\mu }}\mu (S_{i}|\theta^{\mu }),$$

where $\nabla_{\theta_{\mu }}J$ is the gradient.

Update actor target network parameter $\theta_{\mu^{\prime }}$ and critic target network parameter $\theta_{Q^{\prime }}$:

(14) $$\left\{\begin{array}{c} \theta_{Q^{\prime }}\leftarrow \;\tau \theta_{Q}+(1-\tau )\theta_{Q^{\prime }}\;\\ \theta_{\mu^{\prime }}\leftarrow \;\tau \theta_{\mu }+(1-\tau )\theta_{\mu^{\prime }}, \end{array}\right.$$

where the value domain of *τ* is [0, 1].

#### 3.3.2. AUV Path-Planning Model Based on the DDPG Algorithm with Multiple Sensors

As illustrated in Figure 6, the current state of an autonomous underwater vehicle (AUV) serves as an input, which is fed through the DDPG network. The output of the network represents the virtual action of the AUV. Previous actions are sequentially inputted into the computer, and the virtual actions are processed by a Kalman filter to generate the actual actions. These actions are stored in an experience pool and used for training through random sampling. Throughout the interaction with the environment, the DDPG network is continually updated, and the resulting data become more reasonable after undergoing Kalman filtering. Simultaneously, the AUV is in a constant learning process, optimizing subsequent control strategies. The critic component of the neural network consists of two hidden layers with 512 and 256 neurons, while the actor component has a hidden layer with 256 neurons. The output nodes of the hidden layers are activated using the rectified linear unit (ReLU) activation function, and the final layer of the network employs the hyperbolic tangent (tanh) activation function to limit the output range to [−1, 1]. This architecture ensures the neural network’s capability to process information effectively and make appropriate action predictions in the context of DDPG-based control for AUVs.

#### 3.3.3. State Space

An AUV obtains useful information through state space and uses this information to assist in making decisions. In the DDPG framework, the state space is the input of the neural network, which is defined as follows in this experiment:

(15) $$S_{t}=[x_{t},y_{t},z_{t},v_{t},D_{t}],$$

where $x_{t},y_{t},z_{t}$ represent the position of an AUV in Cartesian coordinate space, $v_{t}$ represents speed, and $D_{t}$ represents obstacles as well as target points in the environment.

#### 3.3.4. Action Space

Since the AUV’s movement is accomplished by yaw angle, pitch angle, and speed, the output of the action is defined as follows:

(16) $$a_{t}=[\dot{v_{t}},\alpha_{t},\beta_{t}],$$

where $\dot{v_{t}}$ represents acceleration. $\alpha_{t}$ and $\beta_{t}$ represent yaw and pitch angles, respectively. Considering the mechanical structure of the AUV, the best yaw and pitch angles can only be selected in the AUV performance range.

#### 3.3.5. Reward Function

The reward function is the key of DRL and is used to evaluate the merits of each action. After a series of actions to achieve the goal, the route with the highest cumulative reward is the best route. The most common reward is a sparse reward. The sparse reward is used in this paper. The reward function of this paper is as follows:

(17) $$reward=\left\{\begin{array}{cc} +10 & Reach\;target\;point\\ -10 & Hit\;an\;obstacle\\ \frac{{3dis}_{1}}{{dis}_{2}}-2 & Dangerous\;navigation\\ \frac{{2dis}_{1}}{{dis}_{2}}-1 & Safenavigation, \end{array}\right.$$

where ${dis}_{1}$ is the distance of the AUV to the nearest obstacle and ${dis}_{2}$ is the distance of the AUV to the center of the nearest obstacle. Utilizing the above reward function allows the AUV to navigate as safely as possible.

#### 3.3.6. Mixed Noise

In order to make the search ability of a robot better, it is necessary to add some noise to the output action of the DDPG. Commonly used noise includes Gaussian noise and OU noise; the former produces uncorrelated searches in the time series, while OU noise produces correlated searches in the time series. The next step of OU noise is affected by the previous step, and the formula is as follows:

(18) $$N_{OU}(d_{at})=\theta (\bar{a}-a_{t})dt+\delta dW_{t,}$$

where $a_{t}$ is the action at time *t*, θ is the learning rate of the stochastic process, $\bar{a}$ is the average of the action sampling data, δ is the random weight of the *OU*, and $W_{t}$ is the Wiener process.

Mixed noise formed by Gaussian noise and *OU* noise can better optimize the search strategy. The mixed noise employed in this paper is as follows:

(19) $$a_{t}∼N_{Gaussian}(a_{t}+N_{OU}(d_{at}),var)$$

where var represents the Gaussian variance. As the training volume accumulates, the robot starts to gradually adapt to the environment, so it needs to reduce the search rate. The var needs to be adjusted, so it is defined as var=var∗C and the C is the specified decay rate.

The path-planning method proposed in this paper not only improves the defects of the original algorithm, but also combines with DRL, as well as taking into account the mechanical properties of an AUV, making the generated path safer. The pseudocode for the multisource-data-assisted AUV path-planning method based on the DDPG algorithm is as Algorithm 1:
**Algorithm 1** Multisource-data-assisted AUV path-planning based on the DDPG algorithm1. Randomly initialize critic network $Q(s,a|\theta^{Q})$ and actor $\mu (s|\theta^{\mu })$ with weights $\theta^{Q}$ and $\theta^{\mu }$2. Initialize target network $Q^{\prime }$ and $\mu^{\prime }$ with weights $\theta^{Q^{\prime }}\leftarrow \theta^{Q}$, $\theta^{\mu^{\prime }}\leftarrow \theta^{\mu }$3. Initialize replay buffer R4.   **for** episode = 1, M **do**5.   Initialize a random process N for action exploration6.   Receive initial observation station state $s_{1}$7.   **for** *t* = 1, T **do**8.      Select action $a_{t}=\mu (s_{t}|\theta^{\mu })+N_{t}$ according to the current policy and exploration noise9.      Select virtual actions based on the current strategy and noise10.    The virtual actions is filtered by Kalman filter to generate the corresponding real       action11.    Perform the virtual actions, and get the corresponding reward and the next       position status12.    Execute action $a_{t}$ and observe reward $r_{t}$ and observe new state $s_{t+1}$13.    Store transition $\left(s_{t},a_{t},r_{t},s_{t+1}\right)$ in R14.    Set $y_{i}=r_{i}+\gamma Q^{\prime }(s_{i+1},\mu^{,}(s_{i+1}|\theta^{\mu^{\prime }})|\theta^{Q^{\prime }})$15.    Update critic by minimizing the loss: $L=\frac{1}{N}\sum_{i}{\left(y_{i}-Q(s_{i},a_{i}|\theta^{Q})\right)}^{2}$16.    Update the actor policy using the sampled policy gradient:$\nabla_{\theta_{\mu }}J=\frac{1}{N}\sum_{i}^{N}{\nabla_{a}Q(s,a|\theta^{Q})|}_{s=s_{i},a=\mu (s_{i})}{\nabla_{\theta^{\mu }}\mu (s|\theta^{\mu })|}_{s_{i}}$17.    Update the target networks: $\begin{array}{c} \theta_{Q^{\prime }}\leftarrow \;\tau \theta_{Q}+(1-\tau )\theta_{Q^{\prime }}\;\\ \theta_{\mu^{\prime }}\;\leftarrow \;\tau \theta_{\mu }+(1-\tau )\theta_{\mu^{\prime }} \end{array}$18.**   end for**
19.** end for**


## 4. Simulation Results

To evaluate the effectiveness of the proposed path-planning method, it is compared to the improved ant colony optimization (IACO) algorithm, IAPF-TD3 algorithm, and IAPF-DDPG algorithm in the context of autonomous underwater vehicles (AUVs). The IACO algorithm is an intelligent algorithm known for its strong performance in path-planning tasks, while the TD3 algorithm represents an advanced deep reinforcement learning (DRL) method. The IAPF-TD3 algorithm is a sophisticated path-planning algorithm, making it a suitable choice for comparison purposes. In the experimental setup, an underwater environment of dimensions 10 hm10 hm6 hm (excluding boundaries) is constructed. Spherical and cylindrical obstacles are randomly positioned within this environment. For clarity, the AUV’s left steer angle is assigned a positive value, while the right steer angle is assigned a negative value. Similarly, the AUV’s climb angle is set as positive, and the descent angle is set as negative. The navigation safe distance parameter is set to 0.03 hm (3 m) to ensure safe navigation. Table 1 provides the AUV’s mechanical capacity parameters and hyper-parameters employed during the training process. Through this comprehensive comparison, the proposed path-planning method can be rigorously evaluated in terms of its performance and effectiveness, providing valuable insights for the field of AUV navigation.

**Figure 7:**
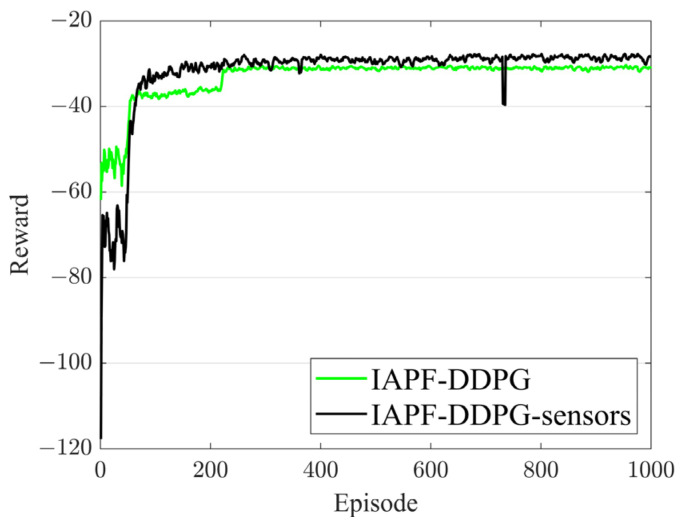
Reward curves of the IAPF-DDPG algorithm and IAPF-DDPG-sensors algorithm.

Figure 7 shows the reward curves of the IAPF-DDPG algorithm and the IAPF-DDPG-sensors algorithm. Figure 7 shows that the proposed algorithm in this paper outperforms the original algorithm in terms of rewards.

In the comparison experiment, in order to intuitively show the experimental results, AUVs equipped with the IACO algorithm, IAPF-TD3 algorithm, and IAPF-DDPG algorithm are all installed with a gyroscope and ranging equipment. These devices do not participate in the operation of the AUVs, only outputting corresponding data for comparison.

**Figure 8:**
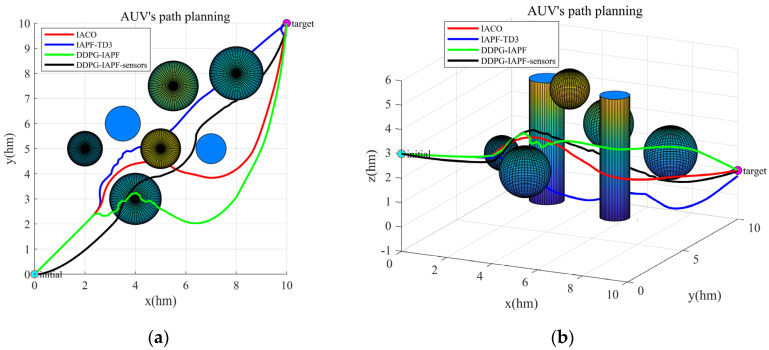
The 3D paths planned by the IACO algorithm, IAPF-TD3 algorithm, IAPF-DDPG algorithm, and the IAPF-DDPG-sensors algorithm. Both the balls and the cylinders represent obstacles. (**a**) Top view. (**b**) 3D view.

Figure 8 illustrates the 3D paths generated by the IACO algorithm, IAPF-TD3 algorithm, IAPF-DDPG algorithm, and IAPF-DDPG-sensors algorithm. Figure 8a presents a top view, while Figure 8b provides a 3D view of the paths. In the figure, the cyan point represents the starting point, and the magenta point represents the target point. The red curve represents the path planned by the IACO algorithm, the blue curve represents the path planned by the IAPF-TD3 algorithm, the green curve represents the path planned by the IAPF-DDPG algorithm, and the black curve represents the path planned by the IAPF-DDPG-sensors algorithm. Figure 8a clearly demonstrates the horizontal projection of the four paths. The red and green curves intelligently choose to bypass complex obstacles, while the blue and black curves navigate between the intricate obstacles. Furthermore, Figure 8a highlights that the red and black curves exhibit minimal fluctuation in vertical height, whereas the blue and green curves display significant variations in numerical height. This comprehensive visualization in Figure 8 provides a comprehensive comparison of the path-planning capabilities of the different algorithms, shedding light on their effectiveness in navigating complex underwater environments.

**Figure 9:**
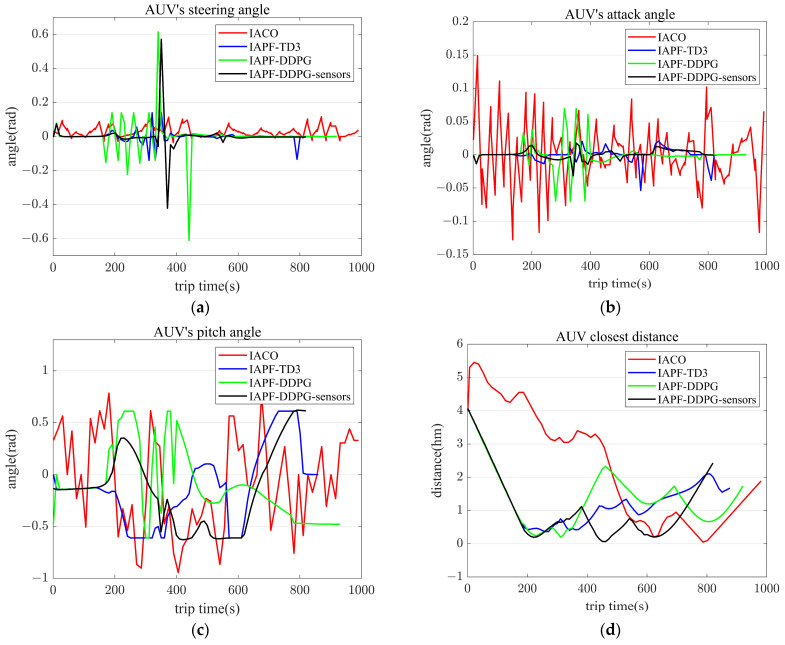
Steering angle, attack angle, pitch angle, and distance from the nearest obstacles of AUVs with four algorithms. (**a**) Steering angle. (**b**) Angle of attack. (**c**) Pitch angle. (**d**) Distance to the nearest obstacle.

Figure 9 provides a comprehensive comparison of the steering angle, attack angle, pitch angle, and closest distance to obstacles for the AUVs, employing the four algorithms. The steering angle represents the horizontal turn angle between consecutive moments, while the attack angle refers to the vertical turn angle. In Figure 9a, a contrast of the steering angle is presented, demonstrating that the proposed algorithm improves the AUV’s steering angle compared to the DDPG-IAPF algorithm. The IACO algorithm consistently maintains a positive steer angle. Figure 9b highlights the comparison of the attack angle, clearly indicating that the proposed algorithm significantly enhances the AUV’s attack angle compared to the other three algorithms. Regarding the pitch angle, as shown in Figure 9c, all four algorithms maintain the AUV’s pitch angle within acceptable performance parameters. Figure 9d visually demonstrates that all four algorithms strive to maintain a safe distance from obstacles to avoid collisions. Notably, the proposed algorithm exhibits a shorter path while ensuring security. The comprehensive analysis provided by Figure 9 underscores the effectiveness of the proposed algorithm in improving steering and attack angles, maintaining appropriate pitch angles, and ensuring collision avoidance. This further establishes its credibility and trustworthiness in terms of AUV navigation in complex underwater environments.

Table 2 shows the experimental data of the steering angle, attack angle, pitch angle, distance to the obstacle (the negative number represents the collision), the path length, and the running time of the AUVs with the four algorithm models.

**Figure 10:**
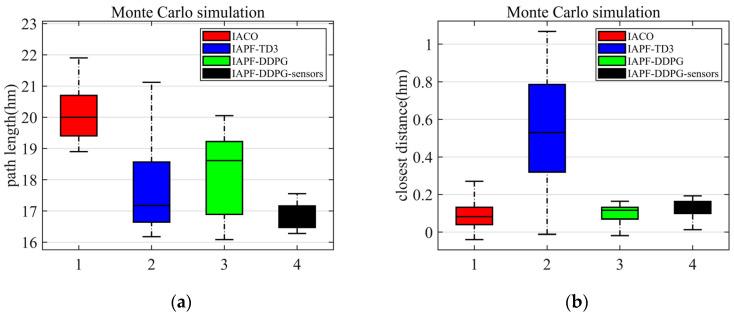
Route lengths and safe distance distribution of the four algorithms in the case of random obstacles. (**a**) The simulation of the path length. (**b**) The simulation of the closest distance.

To verify the reliability of the algorithm, Monte Carlo simulation experiments were performed on the four algorithms. In order to facilitate the comparison of the experimental results on the basis of static obstacles, a certain number of obstacles are selected and allowed to randomly change their position within a specified range, and 1000 simulation experiments were conducted on them. The experimental results are shown in Figure 10, where a is the length of the AUV path and b is the distance between the AUV and the nearest obstacle. It can be seen from Figure 10 that the length of the path generated by the IACO algorithm is longer, while the path generated by IAPF-TD3 algorithm has large fluctuations. The path generated by the proposed algorithm in this paper has small fluctuations and still ensures that AUVs reach their destinations safely. Based on the experimental results, the algorithm proposed in this paper improves the path-planning problem on the premise of ensuring safe navigation. To assess the reliability of the algorithm, Monte Carlo simulation experiments were conducted on the four algorithms. To facilitate a comparative analysis, a predetermined number of obstacles were selected, and their positions were randomly varied within a specified range. A total of 1000 simulation experiments were performed using these dynamic obstacles. The experimental results, depicted in Figure 10, include the length of the AUV path (denoted as “a”) and the distance between an AUV and the nearest obstacle (denoted as “b”). Observing Figure 10, it becomes apparent that the path generated by the IACO algorithm tends to be longer, while the path produced by the IAPF-TD3 algorithm exhibits significant fluctuations. Conversely, the path generated by the proposed algorithm in this paper demonstrates minimal fluctuations while ensuring that AUVs safely reach their destinations. Based on these experimental results, it can be concluded that the algorithm presented in this paper effectively addresses the path-planning problem while maintaining an AUV’s navigation safety. Through rigorous Monte Carlo simulations and analyses, the reliability and robustness of the proposed algorithm are validated, solidifying its efficacy in real-world scenarios.

**Figure 11:**
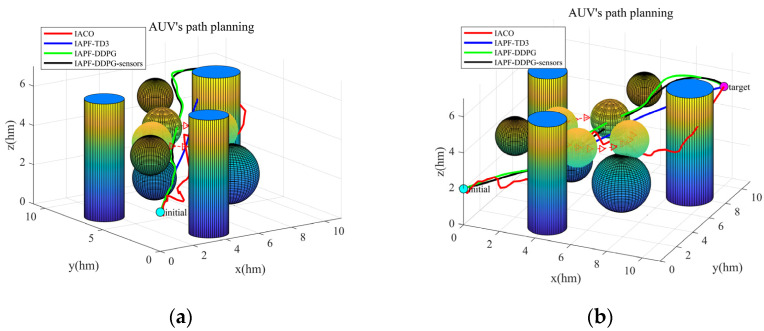
Paths generated by four algorithms in a dynamic environment. Both the balls and cylinders are obstacles, the semi-transparent ball is a dynamic obstacle, and the curve with the red arrow represents the movement path of the dynamic obstacle. (**a**) Left front bottom view. (**b**) Top right front view.

In order to better simulate the underwater driving environment of AUVs, a dynamic simulation environment is also simulated in this paper. Figure 11 shows the path planned by four algorithms in a dynamic environment.

It can be observed from Figure 11 that the path smoothness planned by the IACO algorithm is very low in the face of complex environment; on the other hand, the path generated by the IAPF-TD3 algorithm has good smoothness, but unfortunately it collides with obstacles. The path generated by the IAPF-DDPG algorithm has average smoothness, but it does not collide with an obstacle while reaching the target. The IAPF-DDPG-sensors algorithm optimizes the path on the basis of the IAPF-DDPG algorithm, and causes the smoothness of the path to be improved. Table 3 shows the experimental data, including the steering angle, attack angle, pitch angle, distance from obstacle (negative number indicates collision), path length, and running time of the four algorithm models in a dynamic environment.

**Figure 12:**
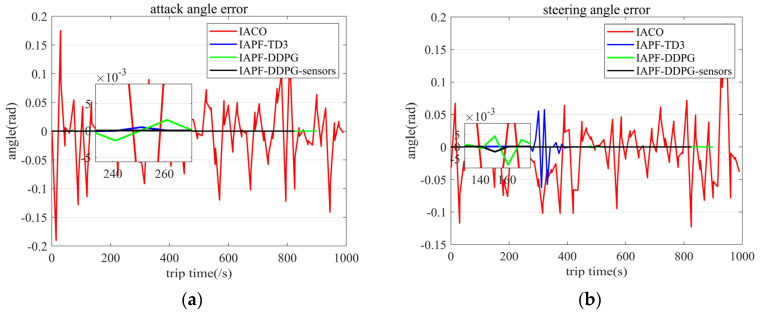
Angle fluctuation caused by measurement error. (**a**) Steering angle error. (**b**) Pitching angle error.

When the AUV moves underwater, the rangefinder has a certain measurement error, and the measurement error of the rangefinder is kept within 0.5%. It is necessary to add random errors to the rangefinder to verify the stability of the generated path of the algorithm. The measurement range of the rangefinder is set at 1 hm, adding random errors to the rangefinder in a static environment, and the error comparison is made through the output of the gyroscope. The experimental results are shown in Figure 12. The experimental results show that the paths generated by the IACO and IAPF-TD3 algorithms have large fluctuations in the presence of measurement errors, while the IAPF-DDPG and IAPF-DDPG-sensors algorithms have strong stability.

In order to estimate the computer resources consumed during the running of the algorithm, the computational complexity needs to be analyzed. The computational complexity only takes into account the operational phase, which is a path-planning task performed on a trained model. Thus, the computational complexity of a DRL-based method is only determined by DNNs. Usually, for dense neural networks, the complexity is $O(\mu \gamma^{2})$, where γ is the number of layers of the neural network and μ is the number of neurons in the widest layer, which is the layer with the largest number of neurons. The number of neurons in each layer depends on the dimension of the input layer, and the number of layers in the neural network is independent of the problem, so the computational complexity of the DRL is $O(n^{2})$.

From the experimental results of the above figures and tables, it can be concluded that the algorithm proposed in this paper makes the path of AUV path-planning shorter, the curve smoother, and its navigation safer in the three-dimensional case. Meanwhile, this method has lower requirements for the steering and climbing of AUVs, which can improve the positioning accuracy of AUVs in real cases.

It is easily seen from the above experimental results that the proposed algorithm is favorable for AUV path-planning in underwater environments. Its advantages are mainly reflected as a smooth trajectory, short length of the planned path (short running time), and small range of course change (easier to achieve heading change and navigation positioning).

## 5. Conclusions and Future Work

Under the condition of simulating the AUV’s operation state deeply, in order to make the AUV’s operation more safe and efficient in the underwater environment, this paper proposes a new algorithm by using the sensor equipment of AUV. The artificial potential field algorithm is improved by using a rangefinder, and an AUV’s course conversion is processed by the Kalman filter algorithm. These were combined with the DDPG algorithm to form the new algorithm proposed in this paper—the IAPF-DDPG-sensors algorithm. Under the condition of ensuring an AUV’s safe navigation, the algorithm uses DDPG network architecture to continuously train and optimize the navigation route. Experiments in static and dynamic environments show that the algorithm has a good effect in underwater environments. The algorithm performs well in terms of the safe travel and path distance of AUVs, and improves the path smoothness (course switching) of AUVs.

In the future, we will focus on the energy optimization and stationary control of AUVs in target-tracking tasks. In addition, we strive to migrate the algorithms mentioned in this paper to the AUV entities, and constantly optimize the path-planning algorithm in this paper in subsequent entity experiments.

## Figures and Tables

**Table 1:** AUV’s capacity parameters and hyper-parameters in training process.

Category	Parameter Name	Parameter Values
**Mechanical capacity**	**V**	**2 m/s (0.02 hm/s)**
	**Ml**	**0.7 rad/s**
	**Mr**	**0.7 rad/s**
	**Mc**	**0.5 rad/s**
	**Md**	**0.5 rad/s**
	**Me**	**1 rad**
	**Mp**	**1 rad**
**Hyper-parameter**	**er**	**2 × 10^6^**
	**bs**	**128**
	**Mi**	**1000**
	**Ms**	**500**
	**Al**	**0.001**
	**Cl**	**0.001**
	**Su**	**0.01**

Abbreviation definitions: V is velocity; Ml is the maximum steering angle (left turn); Mr is the minimum steering angle (right turn); Mc is the maximum attack angle (climbing angle); Md is the minimum attack angle (descent angle); Me is the maximum pitch angle (elevation angle); Mp is the minimum pitch angle (depression angle); er is the experience replay buffer; bs is the batch size; Mi is the max episode; Ms is the max step; Al is the actor learning rate; Cl is the critic learning rate; and su is the soft update rate.

**Table 2:** Experimental data.

Name	IACO	IAPF-TD3	IAPF-DDPG	IAPF-DDPG-Sensors
**Ms**	**0.116**	**0.140**	**0.615**	**0.570**
**Ma**	**0.149**	**0.054**	**0.070**	**0.032**
**Me**	**0.907**	**0.611**	**0.611**	**0.611**
**cd**	**0.049**	**0.365**	**0.199**	**0.064**
**pl**	**19.627**	**17.595**	**18.626**	**16.540**

Abbreviation definitions: Ms is the maximum of the absolute value of the steering angle; Ma is the maximum value of the absolute value of attack angle; Me is the maximum of the absolute value of the climbing angle; cd is the closest distance; and pl is the path length.

**Table 3:** Experimental data.

Name	IACO	IAPF-TD3	IAPF-DDPG	IAPF-DDPG-Sensors
**Ml**	**0.079**	**0.078**	**0.619**	**0.601**
**Mc**	**0.075**	**0.024**	**0.024**	**0.006**
**Me**	**1.086**	**0.462**	**0.611**	**0.498**
**cd**	**−0.818**	**−0.276**	**0.032**	**0.149**
**pl**	**20.692**	**14.740**	**16.009**	**15.772**

Abbreviation definitions: Ms is the maximum of the absolute value of the steering angle; Ma is the maximum value of the absolute value of the attack angle; Me is the maximum of the absolute value of the climbing angle; cd is the closest distance; and pl is the path length.
